# A provably secure coercion-resistant e-voting scheme with confidentiality, anonymity, unforgeability, and CAI verifiability

**DOI:** 10.1371/journal.pone.0324182

**Published:** 2025-06-09

**Authors:** Yun-Xing Kho, Swee-Huay Heng, Syh-Yuan Tan, Ji-Jian Chin

**Affiliations:** 1 Faculty of Information Science and Technology, Multimedia University, Jalan Ayer Keroh Lama, Melaka, Malaysia; 2 School of Engineering, Computing and Mathematics, University of Plymouth, Plymouth, England, United Kingdom; University of Electronic Science and Technology of China, CHINA

## Abstract

Ensuring both cast-as-intended (CAI) verifiability and coercion-resistance in e-voting remains a critical challenge. The e-voting scheme proposed by Finogina and Herranz in 2023 represents the first notable advancement in reconciling these conflicting requirements. CAI verifiability allows voters to confirm that their intended vote has been correctly recorded, even without a secure channel to the election committee, while coercion-resistance prevents external influence and vote-selling. However, essential security properties such as confidentiality, anonymity, unforgeability, and double-voting prevention fall outside the scope of Finogina and Herranz’s e-voting scheme, leaving significant gaps in its security guarantees. To address this limitation, we propose a novel e-voting scheme that simultaneously achieves CAI verifiability, coercion-resistance, confidentiality, anonymity, unforgeability, and double-voting prevention while maintaining an asymptotic complexity of 𝒪(n). To the best of our knowledge, no existing scheme satisfies all these properties concurrently. Moreover, we establish that anonymity inherently implies CAI verifiability in e-voting schemes, a result of independent interest. By strengthening security and privacy guarantees, our work bridges existing gaps and provides a comprehensive security model that serves as a foundation for the design of future e-voting systems.

## 1 Introduction

**Fig 1:**
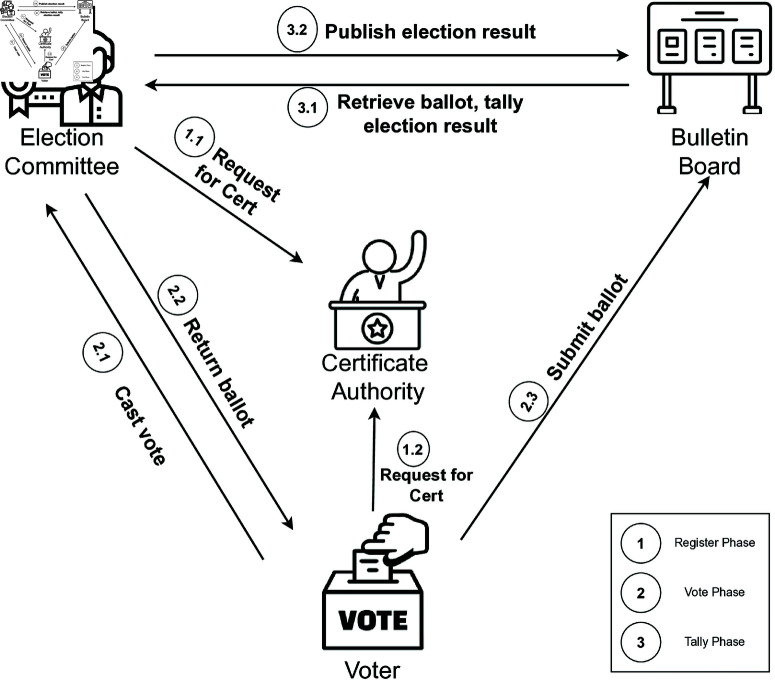
Overview of an e-voting scheme.

The world of electronic voting systems (e-voting) has evolved significantly since it was first introduced by Chaum in 1981. Sections have been renumbered sequentially to maintain order in text, please check and verify.It is a platform designed to facilitate collaborative decision-making and enables voters to cast their votes and the election committee to count ballots electronically. A typical e-voting scheme contains three phases, namely, register, vote, and tally. Several entities are involved in the system, including voters, the election committee, a certificate authority, candidates, and adversaries, as presented in Fig 1. From Fig 1, we can see that during the register phase, both voters and the election committee are registered and obtain certificates from the certificate authority. Additionally, the list of candidates is prepared, and the public parameters of the e-voting system are distributed. The voting phase involves voters casting their votes, while the tallying phase encompasses counting the ballots and announcing the election results.

Several security properties must be in place to ensure the security of an e-voting system. According to Kho *et al*. [1], these essential properties include confidentiality, anonymity, unforgeability, coercion-resistance, and cast-as-intended (CAI) verifiability. Various schemes with security enhancements have been proposed and put into practice [2–9]. These schemes aim to ensure that e-voting is secure and that voters can cast their votes without fear of coercion or privacy violations.

Finogina and Herranz [3] were the first to address the compatibility issue between CAI verification and coercion-resistance. CAI verifiability ensures that voters can verify whether their intended vote has been accurately recorded, even in the absence of a secure channel between voters and the election committee. According to Finogina and Herranz [3], several techniques in the literature aim to achieve CAI verifiability, such as using proofs to verify the cast ballot, tracking numbers, cast-and-audit, voting codes, QR codes, hardware tokens or voting cards with return codes. However, all these techniques often generate some form of evidence or receipt containing voting information for the voter, which directly contradicts the requirement of coercion-resistance, as it must prevent the creation of evidence that could be used to prove a voter’s choice.

In addition to CAI verifiability, the risk of coercion and vote buying is another significant concern. Coercers may attempt to influence how voters cast their ballots, or malicious voters may sell their votes to coercers, undermining the fairness and legitimacy of the electoral process. Therefore, coercion-resistance is a critical property of any e-voting system that aims to ensure the integrity and fairness of the electoral process.

The JCJ protocol proposed by Juels *et al*. in 2005 [5] is a well-known protocol that provides coercion-resistance. The protocol enables voters to deceive coercers by generating fake voting credentials that are indistinguishable from genuine ones. According to Finogina and Herranz [3], existing coercion-resistant e-voting approaches often lack compatibility with CAI verifiability when trusted channels between voters and the election committee are removed. CAI verifiability is another critical property of secure e-voting systems, as it ensures that voters’ intentions are accurately reflected in the final tally. The core challenge in e-voting systems lies in balancing coercion-resistance and CAI verifiability, particularly when encryption is managed by the election committee and secure channels between voters and the election committee are absent. In such a setup, while encryption performed by the election committee ensures that voters cannot access vote verification materials, thereby guaranteeing coercion-resistance, it also raises concerns about whether voters can verify that their ballot is encrypted with their intended vote. Finogina and Herranz therefore addressed this issue by proposing an e-voting protocol that achieves both coercion-resistance and CAI verifiability without relying on secure channels between voters and election authorities.

As noted by Finogina and Herranz, it is reasonable to assume that a coercer cannot maintain continuous control over a voter and would not resort to severe threats, such as threatening the voter with a firearm, as the voter would have no choice but to comply in such cases. If such severe threats were involved, the issue would extend beyond the voting process and fall under the jurisdiction of law enforcement [3]. Therefore, coercion-resistance is meaningful only when the coercer has limited observational capabilities, allowing the voter to resist without incurring significant risks [3].

Consider a common scenario where a voter arrives at the polling station to cast their vote [3]. Could a coercer physically prevent the voter from gaining entry to the polling station? Could they compel the voter to produce a photocopy of their completed ballot as proof of their vote? In the context of e-voting, these concerns translate into questions such as: “Are forced abstention attacks permissible?" and “Can coercers extract vote-verification materials during the voting process?" These questions are critical when evaluating the integrity and security of an e-voting system under potential coercion.

To complicate matters further, some voters with malicious intent may deliberately choose to sell their votes, turning the process into a transaction. Additionally, e-voting introduces a separation between the stages of registration, vote casting, and vote verification, each of which can occur at different times. This separation brings forth a new and significant question: “At precisely which stage can the coercer observe or exert influence over the voter?"

In this work, we adopt the coercion-resistant protocol developed by Finogina and Herranz. In the coercion-resistance setting proposed by Finogina and Herranz, the coercer can communicate with the voter prior to the vote protocol execution and compel the voter to select a particular selection v^*^. During the voting protocol execution, the secure channel between the voter and the election committee is not accessible to the coercer, which is crucial for maintaining meaningful coercion-resistance. However, the coercer can observe the public channel through which the ballot is posted to the bulletin board. After the voting protocol has been executed, the coercer expects to receive *Trc*, which includes the voter’s preference, the corresponding ballot, and the randomness used during the voting process.

To counteract coercion in this scenario, the key idea indicates that the voter should always retain the capability to trick the coercer [3]. The voter can execute the voting protocol with their chosen vote *v* and subsequently replicate all necessary parameters, such as challenges, to convince the coercer that the cast ballot corresponds to v^*^ [3]. The coercer can determine whether the voter adhered to their instructions and cast their vote for v^*^ [3].

Finogina and Herranz [3] addressed the compatibility issue between CAI verifiability and coercion-resistance in the literature. However, their work does not provide rigorous proofs for other essential security properties required by an e-voting system, namely, confidentiality, anonymity, unforgeability, as well as the prevention of double voting [1]. To the best of our knowledge, no existing concrete e-voting scheme simultaneously satisfies confidentiality, anonymity, and unforgeability while also being compatible with CAI verifiability and coercion-resistance.

To address these shortcomings, we propose an e-voting scheme that builds upon the coercion-resistance protocol compatible with CAI verifiability, as introduced by Finogina and Herranz [3]. Our approach enhances their system to ensure it meets the necessary security properties: coercion-resistance, CAI verifiability, confidentiality, anonymity, unforgeability, and prevention of double voting. We further demonstrate that anonymity inherently implies CAI verifiability in e-voting schemes, a result that may be of independent interest. By integrating additional security measures and refining the protocol, our scheme aims to provide a robust solution for secure and verifiable e-voting.

## 2 Related works

In this section, we review coercion-resistant e-voting schemes from the year 2019 till present.

Smyth [10] presented Athena, a verifiable, coercion-resistant e-voting with linear complexity in JCJ setting. Their scheme revealed anonymised credentials to eliminate ballots cast using identical private credential with linear complexity and utilised plaintext equality tests on every mixed ballot individually, containing a mixed public credential and to exclude any unauthorised mixed ballots also with linear complexity. Aranha *et al*. [11] argued that the coercion-resistant protocol proposed by Smyth offered lower security compared to the JCJ scheme, as it revealed the number of votes associated with each credential.

Grontas *et al*. [12] presented the first e-voting scheme that achieved end-to-end verifiability and everlasting privacy in JCJ setting [5]. They proposed a new cryptographic primitive, namely, publicly auditable conditional blind signature where after the interaction, the voter will be issued a token by the signing server, the token can only be verified by the designated verifier. The everlasting privacy intends to protect votes from more powerful adversary (e.g. quantum computing). Their everlasting privacy property is achieved by assuming the existence of an anonymous channel to exchange private data between the election authorities. However, the work did not present a rigorous security analysis.

Estaji *et al*. [13] revisited the JCJ e-voting scheme [5] to enhance its usability and practicality. In particular, they addressed the issue where voters could not directly validate whether their cast ballots were valid and included in the final tally by introducing new methods for duplicate removal within the JCJ framework.

Lueks *et al*. [8] proposed VOTEAGAIN, an e-voting scheme that uses a revoting mechanism to provide coercion-resistance and can handle systems with millions of voters. However, Haines *et al*. [4] argued that VOTEAGAIN is insecure because it relies on a single, completely trusted voting authority to achieve verifiability and coercion-resistance. Furthermore, a malicious bulletin board could compromise the privacy, verifiability, and coercion-resistance of their scheme. Haines *et al*. [4] introduced a variant of VOTEAGAIN that reduces reliance on voting authorities without compromising the efficiency and usability of the original scheme. Their future work involves formally proving that their modifications to VOTEAGAIN maintain the scheme’s security properties.

Rønne *et al*. [14] introduced a quantum-safe, coercion-resistant e-voting scheme within the JCJ framework. They addressed the complexity challenges in the tally phase of the JCJ protocol by employing fully homomorphic encryption (FHE), allowing the tallying process to be executed in linear time while also ensuring quantum safety. It is worth noting that we do not compare the computational cost of this scheme, as it utilises different cryptographic tools. Aranha *et al*. [11] claimed the coercion-resistant protocol introduced by Rønne *et al*. offered lower security compared to the JCJ scheme, as it revealed the number of votes associated with each credential.

Cortier *et al*. [2] identified a major issue with the JCJ protocol: it leaks the total count of received ballots, valid ballots, and revotes during the revoting phase. Hence, they proposed CHide, a new coercion-resistance protocol that solves the leakage issues in JCJ protocol. However, Aranha *et al*. [11] argued that CHide requires a larger asymptotic complexity of $𝒪(n^{2})$. They reduced the CHide’s asymptotic complexity to 𝒪(n log*n*) to speed up the tallying protocol. However, their improved scheme did not consider voter authentication.

Finogina and Herranz [3] proposed the first CAI verifiability and coercion-resistance setting without requiring a secure channel between election authorities and voters. However, their proposed scheme did not claim some important security properties such as confidentiality of ballot, voter’s anonymity, ballot unforgeability and double-voting prevention [1]. The scheme proposed by Finogina and Herranz focuses solely on the voting algorithm and omits the tallying process, as the tally can be conducted using various verifiable methods suggested in the literature, such as verifiable shuffling of ciphertexts, verifiable decryption, and verifiable homomorphic tallying of the final results.

Spadafora *et al*. [15] presented a decentralised e-voting protocol designed to resist coercion and vote-selling while ensuring complete transparency, although it does not achieve receipt-freeness. The protocol leverages blockchain technology to ensure decentralisation and benefits from blockchain’s transparency and non-repudiation properties. The security of the protocol is rigorously proven under the Decisional Diffie-Hellman (DDH) assumption for prime-order cyclic groups and standard blockchain robustness assumptions.

Giustolisi and Garjan [16] introduced an Internet voting scheme that balances efficiency with coercion-resistance. The scheme employs noise ballots to obscure legitimate votes and a cleansing process to eliminate invalid ballots without revealing sensitive information. The scheme supports coercion-resistance, including scenarios with revoting, and enables linear tallying without the use of mixnets or MPC, utilising exponential ElGamal encryption and non-interactive zero-knowledge proofs (NIZKPs).

Chen *et al*. [17] proposed an e-voting scheme aimed to prevent bribery and coercion. By utilising the advantages of a subliminal channel, the channel transmitted a secret message that was modified from the original, ensuring that the receiver only received the general signature without access to the hidden message. Consequently, the e-voting scheme remains secure even if the voting secret is leaked, as the briber or coercer cannot determine the voter’s actual vote. However, the briber or coercer could still attack the channel by preventing the voter’s vote from being sent. To mitigate this risk, Chen *et al*. employed a smart card, which served as a protective mechanism to prevent the subliminal channel from being compromised.

Several recent studies have explored advancements in secure e-voting protocols by leveraging emerging technologies and optimised cryptographic techniques. For instance, Elhabob *et al*. [18,19] proposed equality tests in public key encryption and identity-based encryption with cryptographic reverse firewalls, which could potentially be adapted for detecting duplicate ballots in e-voting while preserving voter privacy. Hadabi *et al*. [20] introduced a proxy re-encryption scheme with plaintext checkable encryption (PCE) for secure data sharing in Industrial IoT. PCE could enhance coercion-resistant e-voting by ensuring ballots are verifiable while safeguarding voter secrecy. Wang *et al*. [21] presented a secure cross-system encrypted data-sharing scheme based on attribute-based encryption (ABE), which could be utilised in e-voting to manage voter authentication and control access to encrypted ballots. Furthermore, Xiong *et al*. [22] proposed a scheme that could contribute to revocable authentication in e-voting, ensuring that voters cannot be linked to their past votes. However, further in-depth research is required to assess the feasibility of fully integrating these techniques into e-voting systems.

### 2.1 Our contributions

We introduce a secure e-voting scheme established on the anonymous authentication proposed by Li *et al*. [7] and Finogina and Herranz’s coercion-resistant voting protocol [3]. The main technical difficulty in this work is to rigorously model and prove the security of confidentiality, anonymity, unforgeability, coercion-resistance, and CAI verifiability, for the proposed scheme. This is a challenging but crucial task that could have laid the security foundation for future e-voting schemes. As an independent interest, we also prove that anonymity implies CAI verifiability for e-voting schemes.

### 2.2 Organisation of this paper

The remainder of this paper is organised as follows. Sect 3 discusses the cryptographic tools underlying our e-voting scheme. Sect 4 defines the security properties and requirements for generic e-voting schemes. In Sect 5, we prove that anonymity implies CAI verifiability within e-voting schemes. Our proposed scheme, along with its security analysis, is presented in Sect 6. Finally, we conclude the paper in Sect 8.

## 3 Cryptographic preliminaries

In this section, we describe the underlying cryptographic tools employed in constructing our e-voting scheme, namely, sigma protocol, commitment scheme, multi-signature, public key encryption (PKE) scheme and event-oriented linkable and traceable anonymous authentication scheme (EOLTAA).

### 3.1 Sigma protocol

A sigma protocol is a 3-move protocol for polynomial time relation, *R*. A sigma protocol Σ=⟨P,V⟩ for *R* runs as follows [23]:

- Commit P(x,w)→a. The prover *P* runs algorithm Commit on common input *x* with corresponding witness *w* to output the first message *a* and submits *a* to the verifier.
- Challenge a→e. Once the verifier *V* receives *a*, chooses a random challenge $e\leftarrow \{0,1{\}}^{l}$ where *l* is the challenge length and submits *e* to the prover.
- Response e→z. Once the prover *P* receives *e*, runs algorithm Response on (*x*,*w*,*e*) to produce *z* and submits *z* to the verifier.

The verifier *V* outputs decision 1 to accept or 0 to reject the transcript (*a*,*e*,*z*) on *x*.

**Definition 1.** *A Sigma protocol for relation R is a 3-move protocol that requires to satisfy the three following properties [24,25]:*

- *Completeness. If* (x,w)∈R*, then all honest 3-move transcripts for* (*x*,*w*) *are always accepted.*
- *Special soundness. There exists a probabilistic polynomial time (PPT) extraction algorithm on input two valid transcripts* (*a*,*e*,*z*) *and* $\left(a,e^{\prime },z^{\prime }\right)$ *for x with* $e\neq e^{\prime }$*, produces a witness w such that* (x,w)∈R.
- *Special honest-verifier zero-knowledge. There exists a PPT simulator that inputs on any instance x and any challenge e, creates a transcript* (*a*,*e*,*z*) *such that the resulting triple is distributed identically to a valid transcript created by an actual protocol execution between the honest prover P*(*x*,*w*) *and the verifier V*(*x*).

### 3.2 Commitment scheme

A commitment scheme consists of three protocols [26]:

- Setup $(1^{\lambda })\to param_{com}$. This protocol takes security parameters $1^{\lambda }$ as input and produces public parameters *param*_*com*_ along with randomness *RS*_*com*_, plaintext *M*_*com*_ and commitment spaces *C*_*com*_.
- Commit $\left(param_{com},e,\hat{r})\to (cmt,d\right)$. This protocol takes *param*_*com*_, a plaintext $e\in M_{com}$ and a randomness $\hat{r}\in RS_{com}$ as input and produces a commitment $cmt\in C_{com}$ and an opening value *d*. The committer submits *cmt* to the verifier. Note that we denote $rand_{v}=(e,\hat{r})=d$ throughout this paper.
- Open (e,cmt,d)→b. This protocol takes (*e*,*cmt*,*d*) as input and produces decision b∈{0,1} where 0 to reject or 1 to accept.

**Definition 2.** *A commitment scheme is required to satisfy the three following properties [25]:*

- *Correctness. A commitment scheme is considered correct if the protocol is executed honestly between the committer and the verifier, the verifier will always accept in the verification phase for all messages that can be committed.*
- *Hiding. A commitment should not reveal any information about e*. *Formally, a commitment is computational hiding if for any PPT* 𝒜*, it contains:*
  $$\mathrm{Pr}\left[\begin{array}{c} b^{\prime }=b: \end{array}\begin{array}{c} pp\leftarrow \text{Setup}(1^{\lambda });\\ (e_{0},e_{1})\leftarrow 𝒜(pp);\\ b\overset{\text{\$}}{\leftarrow }\{0,1\},\hat{r}\overset{\text{\$}}{\leftarrow }R,c\leftarrow \text{Com}(e_{b};\hat{r});\\ b^{\prime }\leftarrow 𝒜(c); \end{array}\right]\leq \frac{1}{2}+\text{negl}(\lambda ).$$
- *Binding. A commitment cannot be opened to two different messages. Formally, a commitment is computational binding if for any PPT* 𝒜*, it contains:*
  $$\mathrm{Pr}\left[\begin{array}{c} e_{0}\neq e_{1}\wedge \\ Com(m_{0};\hat{r_{0}})=Com(m_{1};\hat{r_{1}}): \end{array}\begin{array}{c} pp\leftarrow \text{Setup}(1^{\lambda });\\ (e_{0},r_{0},e_{1},r_{1})\leftarrow 𝒜(pp); \end{array}\right]\leq \text{negl}(\lambda ).$$

### 3.3 Multi-signature

A multi-signature has four algorithms [27]:

- MS.Setup $(1^{\lambda })\to params$. This algorithms takes $1^{\lambda }$ as the input and produces parameters *params* of the signature scheme.
- MS.KeyGen $params\to (pk_{i},sk_{i})$. This algorithms takes *params* as input and produces a set of public and secret keys $\left(pk_{i},sk_{i}\right)$ of signer *i*.
- MS.Sign $(params,sk_{i},m)\to \sigma$. This is an interactive algorithm runs between signers to sign *m*. After a few interactions, the algorithm produces the multi-signature σ.
- MS.Verify $(pk_{i},\sigma ,m)\to 1/0$. This algorithm takes multi public key sets of signer *i*, *m* and σ as input and produces 1 if the σ is valid or 0 if the σ is invalid.

**Definition 3.** *A multi-signature is required to satisfy existential unforgeability under adaptively chosen message attacks (EUF-CMA). The EUF-CMA game is defined as follows [28]:*

- *Register phase: The Challenger generates a target key pair* (pk^*^,sk^*^) of the honest user and passes pk^*^ *to* 𝒜.
- *Training phase:* 𝒜 *can query MS.Sign Oracle by preparing m and* $L=\{pk_{1},...,pk_{n}\}$ of purported signers where pk^*^ *must happen at least once.* 𝒜 can choose the public keys in any manner it desires, including as a function of pk^*^ *and prior protocol interactions.* 𝒜 is able to concurrently initiate the MS.Sign oracle and interact with multiple “clones" of the honest signer. Each clone operates independently, maintaining its own state and randomness, but all share the same keys pk^*^,sk^*^, and follow the signing protocol to generate responses to the messages they receive. Once the honest signer completes its process, the final output is returned to 𝒜 *that whether be a valid multi-signature* σ *or* ⊥.
- *Forging phase:* 𝒜 *outputs* $\left(m^{\prime },\sigma^{\prime }\right)$ *and* $L=\{pk_{1},...,pk_{n}\}$ *and wins the game if the verification of* $(L,m^{\prime },\sigma^{\prime })=1$ *and* $pk^{*}\in L$.

*We say that the multi-signature scheme is EUF-CMA secure if for all PPT* 𝒜 *with negligible advantage:*


$${\text{Adv}}_{𝒜,MS}^{\text{MS-EUF-CMA}}(\lambda ):=\mathrm{Pr}[{\text{MS-EUF-CMA}}_{\text{MS}}^{𝒜}(\lambda )\Rightarrow 1].$$

### 3.4 Public Key Encryption (PKE) scheme

A PKE scheme has three algorithms [29]:

- PKE.KeyGen $\left(1^{k})\to (pk_{e},sk_{e}\right)$. This algorithm takes 1^*k*^ as the input and generates user’s public and private key pair $\left(pk_{e},sk_{e}\right)$.
- PKE.Encrypt $(m,pk_{e})\to C$. This algorithm takes a plaintext message *m* and *pk*_*e*_ as input and generates a ciphertext *C*.
- PKE.Decrypt $(C,sk_{e})\to m$. This algorithm takes a *C* and a *sk*_*e*_ as input and generates the plaintext message *m*.

**Definition 4.** *A PKE scheme has three PPT algorithms* Π=(PKE.KEYGEN,PKE.ENCRYPT, PKE.DECRYPT) *is required to satisfy the two following properties [24]:*

- *Correctness. For each* λ∈ℕ, *each* $\left(sk,pk)\in PKE.KeyGen(1^{\lambda }\right)$, *and each* $m\in \{0,1{\}}^{\lambda }$, *we have* ℙ[PKE.DECRYPT(sk,PKE.ENCRYPT(pk,m))=m]=1.
- *Semantic security. Let* 𝒜 *be the PPT* 𝒜. *Consider the following two experiments:*
  $$\begin{array}{c} {Exp}_{\Pi ,𝒜}^{ind-0}(\lambda ):\\ \left(pk,sk)\leftarrow \$\text{PKE.KEYGEN}(1^{\lambda }\right)\\ \left(m_{0},m_{1})\leftarrow \$𝒜(pk\right)\\ b^{\prime }\leftarrow \$𝒜(PKE.ENCRYPT(pk,m_{0}))\\ \text{return }b^{\prime } \end{array}\begin{array}{c} {Exp}_{\Pi ,𝒜}^{ind-1}(\lambda ):\\ \left(pk,sk)\leftarrow \$\text{PKE.KEYGEN}(1^{\lambda }\right)\\ \left(m_{0},m_{1})\leftarrow \$𝒜(pk\right)\\ b^{\prime }\leftarrow \$𝒜(PKE.ENCRYPT(pk,m_{0}))\\ \text{return }b^{\prime } \end{array}$$
  *We say* Π *is secure if for every PPT* 𝒜 *there exists a negligible function* ν:ℕ→[0,1] *such that:*
  $$|\mathbb{P}[{\text{Exp}}_{\Pi ,𝒜}^{\text{ind}-1}(\lambda )=1]-\mathbb{P}[{\text{Exp}}_{\Pi ,𝒜}^{\text{ind}-0}(\lambda )=1]|\leq \nu (\lambda ).$$

### 3.5 Event-Oriented Linkable and Traceable Anonymous Authentication scheme (EOLTAA)

The EOLTAA Scheme has seven algorithms [7,30]:

- CSetup $\left(1^{k})\to (MPK,MSK\right)$. This algorithm takes 1^*k*^ as input and produces a pair of master public and private key (*MPK*,*MSK*).
- UKeyGen $\left(1^{k})\to (upk,usk\right)$. This algorithm takes 1^*k*^ as input and produces a pair of public and private key (*upk*,*usk*).
- CertGen (MSK,upk)→Cert. This algorithm takes *MSK* and *upk* as input and produces a certificate (*Cert*) analogous to *upk*.
- Auth (m=e∥p,(upk,usk),Cert,MPK)→π. This algorithm takes payload (*p*), *MPK*, message (*m*), event identifier (*e*), (*upk*,*usk*), and *Cert*, as input and produces an authentication token (π) on *m*.
- Verify (m,π,MPK)→0/1. This algorithm takes *m*, π, *MPK* as input to verify the validity of the proof. This algorithm will produce 0 or 1 as the output.
- Link $(m_{1},m_{2},\pi_{1},\pi_{2})\to 0/1$. This algorithm takes two valid *m*, and $\left(m_{1},\pi_{1}),(m_{2},\pi_{2}\right)$ as input and produces 1 if the two *m* are associated with a common event that authenticated with the identical user; else, produces 0.
- Trace $(m_{1},m_{2},\pi_{1},\pi_{2})\to ⟂/upk$. This algorithm takes two valid *m*, and $\left(m_{1},\pi_{1}),(m_{2},\pi_{2}\right)$ as input and produces *upk* of the user who validates two messages associated with a common event. Else, it outputs ⟂.

An EOLTAA scheme is required to satisfy unforgeability, linkability, anonymity, and traceability [7].

**Unforgeability.** The unforgeability of EOLTAA schemes is defined as follows:

1. The Challenger executes CSetup $\left(1^{\lambda }\right)$ to generate (*MPK*,*MSK*) and executes UKeyGen $\left(1^{\lambda }\right)$ to create *n* public private key pairs $\left(upk_{1},usk_{1}),...,(upk_{n},usk_{n}\right)$, and passes $MPK,(upk_{1},...,upk_{n})$ to 𝒜.
2. 𝒜 sends the Challenger a public key $upk_{i}\in \{upk_{1},...,upk_{n}\}$. The Challenger runs CertGen (*upk*_*i*_,*MSK*) to obtain a certificate $\sigma_{i}$.
3. 𝒜 selects and sends *m*_*i*_ and *upk*_*j*_ to the Challenger. 𝒜 requests the Challenger to authenticate *m*_*i*_. The Challenger runs $Auth(m_{i},upk_{j},usk_{j},\sigma_{j},MPK)$ to obtain the authentication token $\pi_{i}$.
4. 𝒜 selects a new m^*^ and upk^*^ and generates $\pi^{*}$. 𝒜 produces $\left(upk^{*},m^{*},\pi^{*}\right)$. 𝒜 wins if the $Verify(m^{*},\pi^{*},MPK)=1$ and (upk^*^,m^*^) is not in the pairs $\left(upk_{j},m_{i}\right)$ that was created during the query phase.
  The success probability of 𝒜 in winning the unforgeability game is ${Adv}_{𝒜}^{Unf}(\lambda )=\mathrm{Pr}[𝒜$ wins the game].

**Definition 5.** *An EOLTAA scheme is unforgeable if for all PPT* 𝒜, ${Adv}_{𝒜}^{Unf}(\lambda )$ *is negligible.*

**Linkability.** The linkability of EOLTAA schemes is defined as follows:

1. The Challenger executes CSetup $\left(1^{\lambda }\right)$ to create (*MPK*,*MSK*) and executes UKeyGen $\left(1^{\lambda }\right)$ to create $\left(upk_{1},usk_{1}),...,(upk_{n},usk_{n}\right)$, and passes $MPK,(upk_{1},...,upk_{n})$ to 𝒜.
2. 𝒜 runs UKeyGen $\left(1^{\lambda }\right)$ to receive (*upk*,*usk*). 𝒜 sends *upk* to the Challenger. The Challenger executes *CertGen*(*upk*,*MSK*) and returns σ.
3. 𝒜 selects and sends *m*_*i*_ and *upk*_*j*_ to the Challenger. 𝒜 requests the Challenger to authenticate *m*_*i*_. The Challenger runs $Auth(m_{i},upk_{j},usk_{j},\sigma_{j},MPK)$ to obtain the authentication token $\pi_{i}$.
4. 𝒜 chooses two $m_{1}^{*}$ and $m_{2}^{*}$ to share a common event and produces two $\pi_{1}^{*}$ and $\pi_{2}^{*}$, respectively. 𝒜 produces $\left(m_{1}^{*},\pi_{1}^{*}\right)$ and $\left(m_{2}^{*},\pi_{2}^{*}\right)$. 𝒜 wins if $Verify(m_{i}^{*},\pi_{i}^{*},MPK)=1$ for i={1,2} and $Link(m_{1}^{*},m_{2}^{*},\pi_{1}^{*},\pi_{2}^{*})=0$.
  The success probability of 𝒜 in winning the linkability game is ${Adv}_{𝒜}^{Link}(\lambda )=\mathrm{Pr}[𝒜$ wins the game].
  **Definition 6.** *An EOLTAA scheme is linkable if for all PPT* 𝒜, ${Adv}_{𝒜}^{Link}(\lambda )$ *is negligible.*

**Anonymity.** The anonymity of EOLTAA schemes is defined as follows:

1. 𝒜 runs CSetup $\left(1^{\lambda }\right)$ to generate (*MPK*,*MSK*) and passes *MPK* to the Challenger.
2. The Challenger executes UKeyGen $\left(1^{\lambda }\right)$ to create $\left(upk_{0},usk_{0}\right)$ and $\left(upk_{1},usk_{1}\right)$, and passes $\left(upk_{0},upk_{1}\right)$ to 𝒜. 𝒜 returns two corresponding $\left(\sigma_{0},\sigma_{1}\right)$ to the Challenger.
3. 𝒜 selects and sends *m*_*i*_ and $upk_{j}\in \{upk_{0},upk_{1}\}$ to the Challenger. 𝒜 requests the Challenger to authenticate *m*_*i*_. The Challenger executes $Auth(m_{i},upk_{j},usk_{j},\sigma_{j},MPK)$ to obtain the authentication token $\pi_{i}$.
4. 𝒜 submits m^*^ to the Challenger that does not belong to the set *m*_*i*_ and the queried messages cannot share a common event. The Challenger selects b∈{0,1}, uses $\left(upk_{b},usk_{b},\sigma_{b}\right)$ to authenticate m^*^ and submits $\pi^{*}$ to 𝒜. 𝒜 then produces his guess $b^{\prime }$. 𝒜 wins if $b^{\prime }=b$.
  The success probability of 𝒜 in winning the anonymity game is ${Adv}_{𝒜}^{Anon}(\lambda )=|\mathrm{Pr}[𝒜$ wins the game] $-\frac{1}{2}|$.
  **Definition 7.** *An EOLTAA scheme is anonymous if for all PPT* 𝒜, ${Adv}_{𝒜}^{Anon}(\lambda )$ *is negligible.*

**Traceability.** The traceability of EOLTAA schemes is defined as follows:

1. The Challenger runs CSetup $\left(1^{\lambda }\right)$ to generate (*MPK*,*MSK*) and executes UKeyGen $\left(1^{\lambda }\right)$ to create $\left(upk_{1},usk_{1}),...,(upk_{n},usk_{n}\right)$, and passes $MPK,(upk_{1},...,upk_{n})$ to 𝒜.
2. 𝒜 runs UKeyGen $\left(1^{\lambda }\right)$ to generate (*upk*,*usk*). 𝒜 sends *upk* to the Challenger. The Challenger executes *CertGen*(*upk*,*MSK*) and returns σ. 𝒜 passes a public key $upk_{i}\in \{upk_{1},...,upk_{n}\}$ to the Challenger. The Challenger runs *CertGen*(*upk*_*i*_,*MSK*) and sends back $\sigma_{i}$ to 𝒜.
3. 𝒜 selects and sends *m*_*i*_ and *upk*_*j*_ to the Challenger. 𝒜 requests the Challenger to authenticate *m*_*i*_. The Challenger runs $Auth(m_{i},upk_{j},usk_{j},\sigma_{j},MPK)$ to obtain the authentication token $\pi_{i}$.
4. 𝒜 chooses $m_{1}^{*}$ and $m_{2}^{*}$ that share a common event and produces two $\pi_{1}^{*}$ and $\pi_{2}^{*}$, respectively. 𝒜 produces $\left(m_{1}^{*},\pi_{1}^{*}\right)$ and $\left(m_{2}^{*},\pi_{2}^{*}\right)$. 𝒜 wins if $Verify(m_{i}^{*},\pi_{i}^{*},MPK)=1$ for i={1,2}; $Link(m_{1}^{*},m_{2}^{*},\pi_{1}^{*},\pi_{2}^{*})=1$ and $Trace(m_{1}^{*},m_{2}^{*},\pi_{1}^{*},\pi_{2}^{*})=⊥/upk^{\prime }$ where $upk^{\prime }\neq upk$.
  The success probability of 𝒜 in winning the traceability game is ${Adv}_{𝒜}^{Trace}(\lambda )=\mathrm{Pr}[𝒜$ wins the game].
  **Definition 8.** *An EOLTAA scheme is linkable if for all PPT* 𝒜, ${Adv}_{𝒜}^{Trace}(\lambda )$ *is negligible.*

## 4 Definition and security requirements for generic e-voting schemes

### 4.1 e-Voting scheme definition

The generic e-voting scheme is composed of three algorithms:

- Register $\left(1^{\lambda })\to \{param,(MPK,MSK),\{(pk_{e},pk_{T}),(sk_{e},sk_{T})\},(upk_{i},usk_{i}),Cert\right\}$: This algorithm is performed by the election committee, voter, and certificate authority. This algorithm takes the $1^{\lambda }$ as the input and produces system public parameters, master key pair (*MPK*,*MSK*), along with two sets of public-private keys for the election committee $\left\{(pk_{e},pk_{T}),(sk_{e},sk_{T})\right\}$ and the voter $\left(upk_{i},usk_{i}\right)$. The voter and election committee then enroll *Cert* with the certificate authority.
- Vote $\left(pk_{e},v,r,\emptyset )↔ElectionCommittee(pk_{e})\to (Bal,Trc,rand_{v}\right)$: This interactive algorithm is executed by the voter and election committee over a secure channel. Voter takes in the election committee’s public key *pk*_*e*_, and his choice of candidate (v,∅). The election committee takes in *pk*_*e*_, voter’s choice of candidates (*v*), and a random value *r*. If the protocol ends successfully, it outputs a ballot *Bal*, *Trc*, $rand_{v}$ and outputs an error ⟂ otherwise.
  We denote *coerc* as an element that is either empty (∅) or a specific instruction *coerc* given to Adversary from the challenger, $rand_{v}$ as the collection of all values deliberately selected by the voter throughout the vote protocol execution, including the challenges and *Trc* as communication transcript between the voter and election committee. *Trc* includes voter’s choice *v* and the ballot *Bal* but for clarity purposes, we still list *Bal* as one of the outputs from voting protocol.
- Tally $(sk_{e},Bal)\to result$: This algorithm is performed by the election committee. The election committee takes *sk*_*e*_, and *Bal* as input, checks validity of the *Bal* and generates the tally result (*result*) of all validated *Bal*.

If the election committee does not send any message to the voter, then it is essentially the same as the non-interactive generic e-voting version introduced by Kho *et al*. [30]. Therefore, the interactive generic e-voting scheme proposed in this section, which considers the election committee, is a generalised version. This generalisation is necessary to capture the coercion-resistance property and CAI verifiability, ensuring that the voter cannot access vote verification materials, as the encryption is performed by the election committee. At the same time, it ensures that an honest voter can verify that his ballot is encrypted with his intended vote.

In this work, we focus solely on the interactive communication between the election committee and the voter throughout the vote protocol execution, ensuring that the encryption of the voter’s vote reflects his intended choice. We leave the challenge of finding a non-interactive voting protocol that also possesses coercion-resistance as an open problem.

### 4.2 Security requirements for e-voting

As outlined in the previous section, our scheme definition diverges from that of Kho *et al*. [30] only in the vote algorithm. In our scheme, we consider an interactive voting process, allowing for communication between the voter and the election committee during the vote submission. In contrast, Kho *et al*.’s scheme follows a non-interactive model, where the voter independently encrypts and submits their ballot without interaction. Despite this key difference, we adopt Kho *et al*.’s broader security definition, making amendments solely to the voting-related components to accurately capture the interactive nature of our scheme.

1. **Confidentiality**. The confidentiality ensures that the ballot is confidential to all parties, except when the election results disclose the vote [30]. The following game demonstrates the indistinguishability under chosen ballot attack (IND-CBAA) security notion for an e-voting scheme. We describe the game between the PPT 𝒜 and Challenger as follows:
  **Algorithm 1. e-Voting Confidentiality Game**.

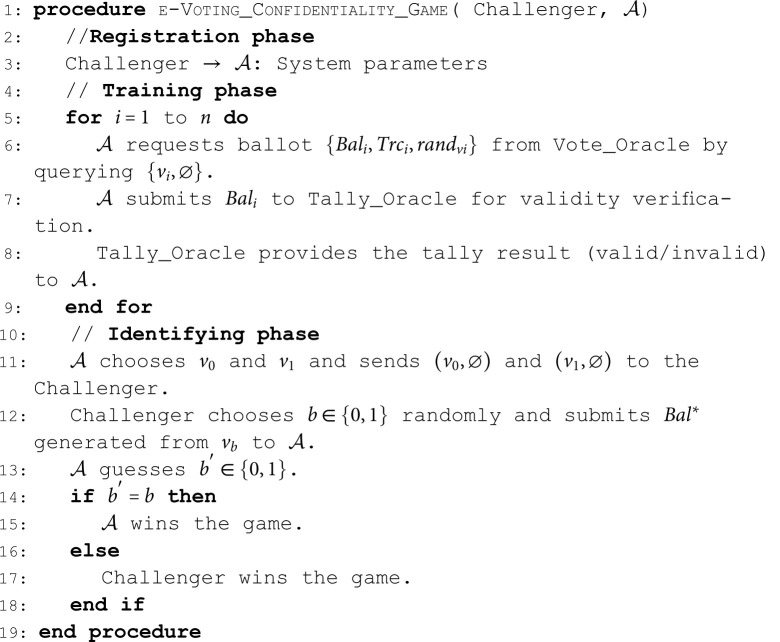

  **Definition 9** (IND-CBAA). *An e-voting scheme is* (ε,t)*-IND-CBAA if no PPT* 𝒜 *can win the game above in time t with an advantage* $\mathrm{Pr}[b^{\prime }=b]\leq \frac{1}{2}+\varepsilon$.
2. **Anonymity**. The anonymity ensures that the identification of the voter remains secret [30]. The following game demonstrates indistinguishability under chosen voter’s vote attack (IND-CVA) security notion for an e-voting scheme. We describe the game between 𝒜 and the Challenger as follows:
  **Algorithm 2. e-Voting anonymity game.**.

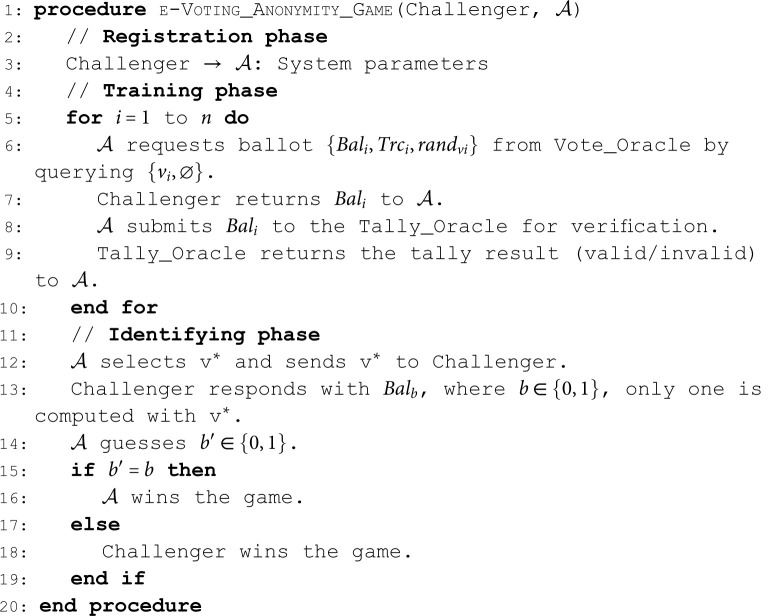

  **Definition 10** (IND-CVA). *An e-voting scheme is* (ε,t)*-IND-CVA if no PPT* 𝒜 *can win the game above in time t with an advantage* $\mathrm{Pr}[b^{\prime }=b]\leq \frac{1}{2}+\varepsilon$.
3. **Unforgeability**. The unforgeability means the inability to forge a valid ballot intended for another voter [30]. The following game demonstrates existential unforgeability under chosen vote attack (EUF-CVA) security notion for an e-voting scheme. We describe the game between 𝒜 and the Challenger as follows:
  **Algorithm 3. e-Voting unforgeability game.**

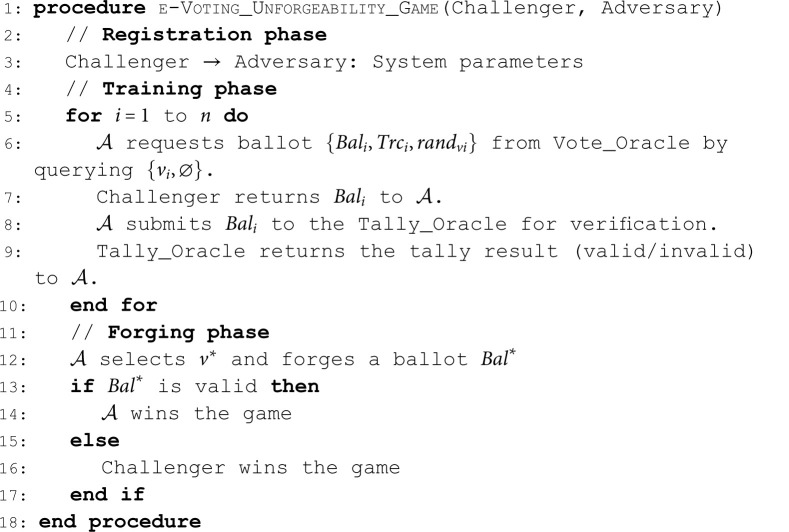

  **Definition 11** (EUF-CVA). *An e-voting scheme is* (ε,t)*-EUF-CVA if no PPT* 𝒜 *can win the game above in time t with an advantage* $\mathrm{Pr}[Bal^{*}\;is\;valid]\leq \varepsilon$.
4. **Coercion-Resistance**. According to Kho *et al*. [1], coercion-resistance in an e-voting scheme means the coercers cannot insist that voters vote in a certain way and the voter cannot prove his vote to the information buyer. We describe the game between 𝒜 and the Challenger as follows:
**Algorithm 4. e-Voting coercion-resistance game.**

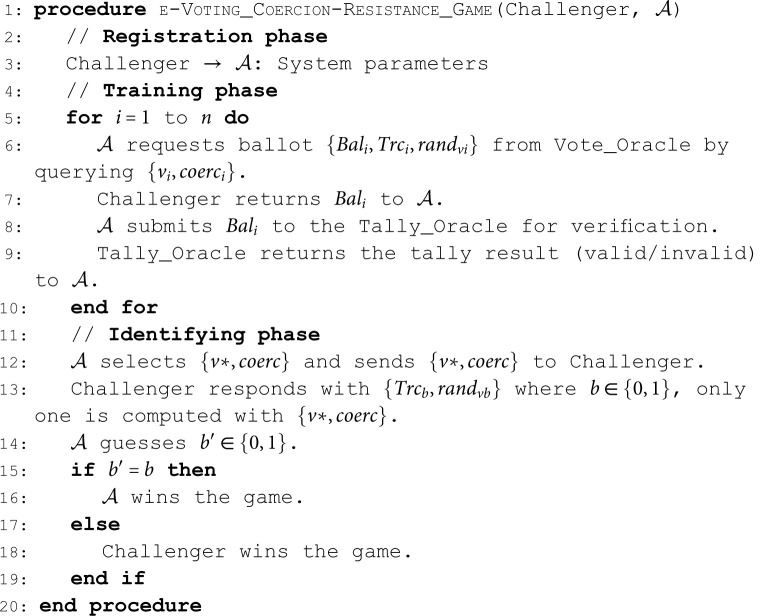

  **Definition 12** (Coercion-Resistance). *An e-voting scheme is* (ε,t)*-coercion-resistance if no PPT* 𝒜 *can win the game above in time t with an advantage* $\mathrm{Pr}[b^{\prime }=b]\leq \frac{1}{2}+\varepsilon$.
5. **Cast-As-Intended (CAI) Verifiability**. According to Finogina and Herranz [3], CAI verifiability in an e-voting scheme guarantees that the malicious election committee cannot deceive the voter by sending an encryption of a voting option different from the one the voter selected to the bulletin board. In other words, the honest voter (not coerced) can verify whether his ballot contains the encryption of his vote. Thus, this property is formalised by considering a dishonest election committee that attempts to deceive the voter by sending the ballot to the bulletin board, which decrypts to v ′ ≠ v *. We define the following game as indistinguishability under chosen voter’s cast vote attack (IND-CAI). We describe the game between 𝒜 and the Challenger as follows:
**Algorithm 5. e-Voting CAI verifiability game.**

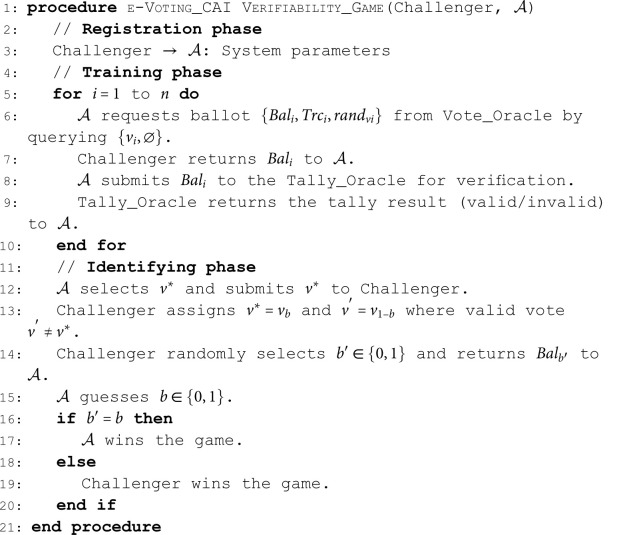

  **Definition 13** (Cast-As-Intended Verifiability). *An e-voting scheme is* (ε,t)*-indistinguishability under chosen voter’s cast vote attack (IND-CAI) if no PPT* 𝒜 *can win the game above in time t with an advantage* $\mathrm{Pr}[b=b^{\prime }]\leq \frac{1}{2}+\varepsilon$.

## 5 Anonymity implies CAI verifiability

We notice that the security game of anonymity and CAI verifiability have high similarities, this motivates us to demonstrate the relationships between the security game of anonymity and CAI verifiability. The one-way equivalency demonstrates if the e-voting scheme possesses anonymity (IND-CVA), then it also possesses CAI verifiability (IND-CAI). The following theorem presents the one-way equivalency between the security game of anonymity and CAI verifiability.

**Theorem 1.** *If an e-voting scheme possesses anonymity (IND-CVA), then it also possesses CAI verifiability (IND-CAI).*

*Proof*: Assume *A*_2_ is an Adversary who $\left(t,q_{a},\varepsilon \right)$-compromises the IND-CAI security of the e-voting scheme and *A*_1_ is the Adversary who $\left(t^{\prime },q_{v},\varepsilon^{\prime }\right)$-compromises the IND-CVA security of e-voting scheme. We aim to demonstrate that e-voting scheme is not $\left(t^{\prime },q_{v},\varepsilon^{\prime }\right)$-secure by showing how *A*_1_ can utilise *A*_2_ to $\left(t^{\prime },q_{v},\varepsilon^{\prime }\right)$-compromise the IND-CVA security of the e-voting scheme. Here, $q_{v}$ represents the number of vote queries, *q*_*a*_ represents the number of authentication queries, ε represents the non-negligible advantage in breaking the IND-CVA in e-voting, $\varepsilon^{\prime }$ represents the non-negligible advantage in breaking IND-CAI in the e-voting, and *t* represents the attack completion time. In this scenario, *A*_1_ can run *A*_2_ as a subroutine while simulating its attack environment.

The CVA Challenger gives *Params* to *A*_1_. *A*_1_ gives *Params* to *A*_2_ and completes the Registration phase. During the Training phase, *A*_2_ sends a vote query {v,∅} to *A*_1_. *A*_1_ submits *v* to Vote oracle via a vote query to generate $\left\{Bal,Trc,rand_{v}\right\}$ and returns $\left\{Bal,Trc,rand_{v}\right\}$ to *A*_2_. *A*_2_ submits a tally query *Bal* to *A*_1_. *A*_1_ submits *Bal* to Tally oracle to check its validity. The Tally oracle provides the tally result to *A*_1_ and *A*_1_ retrieves the validation result from the tally result. *A*_1_ then sends the validation result whether it is invalid or valid to *A*_2_.

Eventually, *A*_2_ concludes the Training phase and initiates the Identification phase. During this phase, *A*_2_ issues v^*^ to *A*_1_. *A*_1_ assigns v^*^ to Vote oracle to generate *C*_*b*_ and *C*_1−*b*_ where b∈{0,1} and one of them is created with v^*^. *A*_1_ generates $\pi_{b}$ on *C*_*b*_ and $\pi_{1-b}$ on *C*_1−*b*_. *A*_1_ sets $Bal_{b}=\{C_{b},\pi_{b}\}$ and $Bal_{1-b}=\{C_{1-b},\pi_{1-b}\}$. *A*_1_ randomly guesses $b^{\prime }\in \{0,1\}$ and delivers $Bal_{b^{\prime }}$ as the IND-CAI challenge to *A*_2_. *A*_2_ outputs its guess $b^{\prime \prime }$ and *A*_1_ uses *A*_2_’s answer as its guess.

Now, we analyse the probability of *A*_1_ winning the IND-CVA game. If the correct guess is *b*, we have:

$$Pr[b^{\prime \prime }=b^{\prime }\;|\;b^{\prime }=b]\leq \frac{1}{2}(\frac{1}{2}+\varepsilon )$$ (1)

When $b^{\prime }=b$, we have $b^{\prime \prime }=b^{\prime }\Leftrightarrow b^{\prime \prime }=b$; this implies:

$$Pr[b^{\prime \prime }=b\;|\;b^{\prime }=b]\leq \frac{1}{4}+\frac{1}{2}\varepsilon$$ (2)

On the other hand, if the correct guess is 1–*b*, we have:

$$Pr[b^{\prime \prime }=b^{\prime }\;|\;b^{\prime }=1-b]\leq \frac{1}{2}(\frac{1}{2}+\varepsilon )$$ (3)

which gives:

$$Pr[b^{\prime \prime }=1-b\;|\;b^{\prime }=1-b]\leq \frac{1}{4}+\frac{1}{2}\varepsilon$$ (4)

Combining (2) and (4), we obtain:

$$Pr[b^{\prime \prime }=b\;|\;b^{\prime }=b]+Pr[b^{\prime \prime }=1-b\;|\;b^{\prime }=1-b]\leq \frac{1}{2}+\varepsilon$$ (5)

Given that *A*_1_ accurately simulates the environment, we conclude that $\varepsilon =\varepsilon^{\prime }$ and $t=t^{\prime }$, as required. In this setup, *A*_1_ runs in time *t*, while *A*_2_ runs in time $t^{\prime }$. ◻

## 6 Our proposed scheme

### 6.1 Scheme construction

Let PKE={PKE.KeyGen,PKE.Encrypt,PKE.Decrypt} represent a semantically secure public key encryption scheme, $\Phi =\{\Phi_{\cdot }CSetup,\Phi_{\cdot }UKeyGen,\Phi_{\cdot }CertGen,\Phi_{\cdot }Auth,\Phi_{\cdot }Verify,\Phi_{\cdot }Link,$ $\Phi_{\cdot }Trace\}$ represent the EOLTAA scheme, MS={MS.Setup,MS.KeyGen,MS.Sign,MS.Verify} represent the secure multi-signature scheme, Σ−protocol={Commit,Challenge,Response} represent the secure Σ-protocol and Commitment={Setup,Commit,Open} represent the secure commitment scheme. Our e-voting scheme contains three algorithms, that is, Register, Vote, and Tally. Note that the bulletin board implemented in our scheme is publicly readable, append-only storage and its contents cannot be altered or forged by any party.

**Register( $1^{\lambda })\to \{param,(MPK,MSK),\{(pk_{e},pk_{T}),(sk_{e},sk_{T})\},(upk,usk),Cert$**}. Election committee runs *MS*.*Setup* to generate public parameters, *params*_*MS*_. Election committee runs setup algorithm in the Commitment to produce *param*_*com*_ along with $M_{com},RS_{com},C_{com},$ where *param*_*com*_ is the public parameter, *M*_*com*_ is the plaintext, *RS*_*com*_ is the randomness, *C*_*com*_ is the commitment spaces. Suppose that the Σ-protocol <*P*,*V*> for the Relation *R*_*e*_ consists of the challenge space Chl $\subset M_{com}$. All these public parameters (*param*) are sent to the bulletin board.

Certificate authority utilises Φ.CSetup to create master public key *MPK* and master private key *MSK*. Election committee runs *PKE*.*KeyGen* to generate $\left(pk_{e},sk_{e}\right)$ where *pk*_*e*_ is the public key, *sk*_*e*_ is the secret key. Voters and the election committee create their set of keys with $\Phi_{\cdot }UKeyGen$ and register *Cert* with the certificate authority. Certificate authority checks the eligibility of the voter and election committee. If the voter and the election committee are eligible, certificate authority will return a certificate to the voter and the election committee where the election committee holds $\left\{(pk_{T},sk_{T}),Cert_{T}\right\}$ and the voters holds $\left\{(upk_{i},usk_{i}),Cert_{i}\right\}$. The election committee uses *params*_*MS*_ as input to the *MS*.*KeyGen* to receive their public key and secret key for signing.

**Vote $\left(pk_{e},v,r,\emptyset )↔ElectionCommittee(pk_{e})\to (Bal,Trc,rand_{v}\right)$**. The vote protocol comprising four communication rounds between the voter and the election committee, operates as follows:

1. The election committee selects a random value *vid* as ID of the e-voting. The election committee generates $\pi_{T}$ to validate *vid* where $\pi_{T}=\Phi_{\cdot }Auth(vid||pk_{T},sk_{T},Cert_{T},MPK)$. Election committee sends $\left(vid||\pi_{T}\right)$ to the bulletin board.
2. Once the voter receives this voting, the voter selects $e\in Chl,\hat{r}\in RS_{com}$ and one candidate *v*, computes a commitment *cmt* and sends (*v*,*cmt*) to the election committee.
3. If *v* is not a valid voting option, the election committee aborts. Else election committee jointly chooses a random value *r*, uses *pk*_*e*_ to jointly encrypt (*v*,*r*) to obtain *C*_*i*_. The election committee then runs *MS*.*Sign* to produce signature σ on the *C*_*i*_. This is to demonstrate that a group of *n* signers jointly create a σ for (*v*,*r*) in such a way that σ persuades the voter that all *n* signers have jointly signed (*v*,*r*). Then it executes first round of the Σ-protocol using the public input $x=(v,C_{i})$ and witness *w* = *r* to create value *a*. The election committee issues $\left(C_{i},\sigma ,a\right)$ to the voter.
4. The voter replies $rand_{v}=(e,\hat{r})$ to the election committee.
5. The election committee verifies if e∈Chl, if $\hat{r}\in RS_{com}$ and if *cmt* is valid. If the verification fails, the election committee aborts. Else, the election committee utilises challenge *e* to produce value *z*, and transmits *z* to voter.
6. Voter runs *MS*.*Verify* to verify if the σ is valid. Voter accepts the interaction if and only if the transcript (*a*,*e*,*z*) is valid on the public input $x=(v,C_{i})$ and σ is a valid signature. If the voter accepts the interaction, voter produces $\pi_{i}=\Phi_{\cdot }Auth(vid||C_{i},upk_{i},usk_{i},Cert_{i},MPK)$. Voter sends $Bal=(C_{i},\pi_{i})$ to the bulletin board.

Note that we denote $Trc=(v,C,a,e,z,cmt,\hat{r})$ and $rand_{v}=(e,\hat{r})$.

**Tally $(sk_{e},Bal)\to result$.** The election committee runs $\Phi_{\cdot }Verify(vid||C_{i},\pi_{i},MPK)$ to verify every received ballot along with its authentication pair. Any invalid ballot is discarded. Then, verify if the valid ballot is being double-vote before by executing $\Phi_{\cdot }Link(C_{i},C_{*},\pi_{i},\pi_{*})$ for every $\left(C_{*},\pi_{*}\right)$ that has been utilised before, and execute $\Phi_{\cdot }Trace(C_{i},C_{*},\pi_{i},\pi_{*})$ identify the identification of the double-vote voter.

The election committee then jointly decrypts all valid ballots using (*sk*_*e*_) and computes the final election result (*result*). The election committee creates zero-knowledge proof $\pi_{result}$ using *sk*_*e*_. Finally, the election committee submits $\left\{result,\pi_{result}\right\}$ to the bulletin board and the final election result is publicly verifiable.

### 6.2 Security analysis

We present a security analysis to demonstrate that the proposed e-voting scheme satisfies the relevant security requirements outlined in Theorems 2 to 6, specifically confidentiality, anonymity, unforgeability, coercion-resistance, and CAI verifiability, as follows:

Confidentiality: If the underlying PKE scheme satisfies IND-CCA, then our proposed e-voting scheme ensures confidentiality. Anonymity: If the underlying anonymous authentication scheme (EOLTAA) is anonymous, then our proposed e-voting scheme guarantees anonymity. Unforgeability: If the underlying authentication scheme (EOLTAA) is unforgeable, then our proposed e-voting scheme upholds unforgeability. Coercion-resistance: If the underlying PKE scheme satisfies IND-CCA, the commitment scheme satisfies the binding property, and the sigma protocol satisfies the special honest-verifier zero-knowledge property, then our proposed e-voting scheme ensures coercion-resistance. CAI Verifiability: If our proposed e-voting scheme ensures anonymity, as demonstrated in Theorem 2, it also guarantees CAI verifiability, as demonstrated in Theorem 1.

Note that our scheme does not require a direct proof (i.e., a detailed proof) because its security follows from our previous work on the transformation framework from e-voting to e-cheque [30]. The key benefit of the proposed transformation framework is that it significantly reduces the complexity of proving security. Specifically, by leveraging the security relationships among e-voting, e-auction, e-cheque, and e-cash in terms of security definitions and requirements, we can derive new schemes from existing ones without starting from scratch, with the assurance that the transformed scheme inherits the security guarantees of the underlying scheme or building block.

#### 6.2.1 Confidentiality.

**Theorem 2.** *Let APKE =* {*Setup, Identification, Validation*} *represent the secure EOLTAA scheme and PKE scheme. Let e-voting = {Register, Vote, Tally} represent the e-voting scheme. If the underlying APKE scheme is* $\left(t,q_{a},\varepsilon \right)$*-indistinguishability under chosen-ciphertext attacks (IND-CCA), then the e-voting scheme is* $\left(t^{\prime },q_{v},\varepsilon^{\prime }\right)$*-IND-CBAA, where*

t=t′,qa=qv,ε=ε′≤12+n(1λ) (6)

*Here,* $q_{v}$ *represents the number of vote queries, q*_*a*_ represents *the number of authentication queries,* ε *represents the non-negligible advantage in breaking IND-CCA in APKE,* $\varepsilon^{\prime }$ *represents the non-negligible advantage in breaking IND-CBAA in e-voting, n represents a negligible function parameterised by* $1^{\lambda }$, and *t represents the attack completion time.*

*Proof*: Assume *A*_2_ is an Adversary who $\left(t^{\prime },q_{v},\varepsilon^{\prime }\right)$-compromises the IND-CBAA security of the e-voting scheme, and $A_{1}=A_{APKE}$ is the Adversary who $\left(t,q_{a},\varepsilon \right)$-compromises the IND-CCA security of the APKE scheme. We aim to demonstrate that APKE scheme is not $\left(t,q_{a},\varepsilon \right)$-secure by showing how *A*_1_ can utilise *A*_2_ to $\left(t,q_{a},\varepsilon \right)$-compromise the IND-CCA security of APKE. In this scenario, *A*_1_ runs *A*_2_ as a subroutine while simulating its attack environment.

The APKE Challenger gives (*Params*,*upk*,*usk*,*Cert*) to *A*_1_. These keys and certificate (*upk*,*usk*,*Cert*) are generated from the EOLTAA scheme. We allow *A*_1_ to possess the (*upk*,*usk*,*Cert*) of the EOLTAA scheme, enabling it to simulate the Vote and Tally oracles for *A*_2_. Although *A*_1_ has access to (*upk*,*usk*,*Cert*) from the EOLTAA scheme, this does not provide an advantage in breaking the IND-CCA security. After this, *A*_1_ gives *Params* to *A*_2_ and completes the Registration phase.

During the Training phase, *A*_2_ sends a vote query {v,∅} to *A*_1_ which represents the Vote oracle from *A*_2_’s perspective. Here, *A*_1_ takes m=(v,∅), encrypts *m* to generate $\left(Trc,rand_{v}\right)$ to obtains *C*, and creates π for *C*. *A*_1_ then returns the result $\{Bal,Trc,rand_{v}\}=\alpha$ to *A*_2_. After that, *A*_2_ submits a tally query as *Bal* to *A*_1_. At this point, *A*_1_ assigns α=Bal and uses the Decrypt oracle to simulate the tallying process for *A*_2_. Specifically, *A*_1_ passes α to the Decrypt oracle to check its validity. The oracle provides the decryption result and *A*_1_ retrieves the validation result from the decryption result and returns the validation result whether it is invalid or valid to *A*_2_.

Eventually, *A*_2_ concludes the Training phase and initiates the Identification phase. During this phase, *A*_2_ selects $\left(v_{0},\emptyset \right)$ and $\left(v_{1},\emptyset \right)$ and gives them to *A*_1_. *A*_1_ assigns $m_{0}=(v_{0},\emptyset ),m_{1}=(v_{1},\emptyset )$ and randomly chooses a bit b={0,1}. *A*_1_ generates $\left(Trc_{b},rand_{vb}\right)$ and obtains *C*_*b*_ from *m*_*b*_ and creates $\pi_{b}$ for *C*_*b*_. *A*_1_ then assigns $Bal_{b}=\{C_{b},\pi_{b}=\alpha_{b}\}$. *A*_1_ delivers *Bal*_*b*_ as the challenge in IND-CBAA to *A*_2_. With a probability $\varepsilon^{\prime }\leq \frac{1}{2}\;+\;n(k)$, *A*_2_ guesses the correct value of $b^{\prime }$. *A*_1_ takes *A*_2_’s guess as its own guess. Since $b^{\prime }=b$, *A*_1_ successfully compromises the IND-CCA security.

Given that *A*_1_ accurately simulates the environment, we conclude that $\varepsilon =\varepsilon^{\prime }$ and $t=t^{\prime }$, as required. In this setup, *A*_1_ runs in time *t*, while *A*_2_ runs in time $t^{\prime }$. ◻

#### 6.2.2 Anonymity.

**Theorem 3.** *Let APKE = {Setup, Identification, Validation} represent the secure EOLTAA scheme and PKE scheme. Let e-voting = {Register, Vote, Tally} represent the e-voting scheme. If the underlying APKE is* $\left(t,q_{a},\varepsilon \right)$*-anonymous, then the e-voting scheme is* $\left(t^{\prime },q_{v},\varepsilon^{\prime }\right)$*-IND-CVA, where*

t=t′,qa=qv,ε=ε′≤12+n(1λ) (7)

*Here,* $q_{v}$ *represents the number of vote queries, q*_*a*_ *represents the number of authentication queries,* ε *represents the non-negligible advantage in breaking the anonymity in APKE,* $\varepsilon^{\prime }$ *represents the non-negligible advantage in breaking IND-CVA in e-voting, n represents a negligible function parameterised by* $1^{\lambda }$, *and t represents the attack completion time.*

*Proof*: Assume *A*_2_ is an Adversary who $\left(t^{\prime },q_{v},\varepsilon^{\prime }\right)$-compromises the IND-CVA security of the e-voting scheme, and $A_{1}=A_{APKE}$ is the Adversary who $\left(t,q_{a},\varepsilon \right)$-compromises the anonymity security of the APKE scheme. We aim to demonstrate that APKE scheme is not $\left(t,q_{a},\varepsilon \right)$-secure by showing how *A*_1_ can utilise *A*_2_ to $\left(t,q_{a},\varepsilon \right)$-compromise the anonymity security of APKE scheme. In this scenario, *A*_1_ runs *A*_2_ as a subroutine while simulating its attack environment.

The APKE Challenger gives $\left(Params,pk_{e},sk_{e}\right)$ to *A*_1_. These keys $\left(pk_{e},sk_{e}\right)$ are generated from the PKE scheme. We allow *A*_1_ to possess the $\left(pk_{e},sk_{e}\right)$ of the PKE scheme, enabling it to simulate the Vote and Tally oracles for *A*_2_. Although *A*_1_ has access to $\left(pk_{e},sk_{e}\right)$ from the PKE scheme, this does not provide an advantage in breaking the anonymity security. After this, *A*_1_ gives *Params* to *A*_2_ and completes the Registration phase.

During the Training phase, *A*_2_ sends a vote query {v,∅} to *A*_1_ which represents the Vote oracle from *A*_2_’s perspective. Here, *A*_1_ takes m=(v,∅), encrypts *m* to generate $\left(Trc,rand_{v}\right)$ to obtains *C*, and creates π for *C*. *A*_1_ then returns the result $\{Bal,Trc,rand_{v}\}=\alpha$ to *A*_2_. After that, *A*_2_ submits a tally query as *Bal* to *A*_1_. At this point, *A*_1_ assigns α=Bal and uses the Decrypt oracle to simulate the tallying process for *A*_2_. Specifically, *A*_1_ passes α to the Decrypt oracle to check its validity. The oracle provides the decryption result and *A*_1_ retrieves the validation result from the decryption result and returns the validation result whether it is invalid or valid to *A*_2_.

Eventually, *A*_2_ concludes the Training phase and initiates the Identification phase. During this phase, *A*_2_ issues v^*^ to *A*_1_. *A*_1_ assigns $m^{*}=v^{*}$ and obtains *C*_*b*_ by encrypting m^*^ where b∈{0,1}. *A*_1_ creates $\pi_{b}$ for *C*_*b*_. *A*_1_ then assigns $Bal_{b}=\{C_{b},\pi_{b}\}=\alpha_{b}$. *A*_1_ delivers *Bal*_*b*_ as the challenge in IND-CVA to *A*_2_. With a probability ε ′ ≤ 1 2 + n ( k ), *A*_2_ correctly guesses b ′. *A*_1_ takes *A*_2_’s guess as its own guess. Since b′ = b, *A*_1_ successfully compromises the anonymity security.

Given that *A*_1_ accurately simulates the environment, we conclude that ε = ε ′ and t ′, as required. In this setup, *A*_1_ runs in time *t*, while *A*_2_ runs in time t ′. ◻

#### 6.2.3 Unforgeability.

**Theorem 4.** *Let APKE = {Setup, Identification, Validation} represent the secure EOLTAA scheme and PKE scheme. Let e-voting = {Register, Vote, Tally} represent the e-voting scheme. If the underlying APKE is* $\left(t,q_{a},\varepsilon \right)$*-unforgeable, then the e-voting scheme is* $\left(t^{\prime },q_{v},\varepsilon^{\prime }\right)$*-EUF-CVA, where*

t=t′,qa=qv,ε=ε′≤12+n(1λ) (8)

*Here,* $q_{v}$ *represents the number of vote queries, q*_*a*_ *represents the number of authentication queries,* ε *represents the non-negligible advantage in breaking the anonymity in APKE,* $\varepsilon^{\prime }$ *represents the non-negligible advantage in breaking EUF-CVA in e-voting, n represents a negligible function parameterised by* $1^{\lambda }$, *and t represents the attack completion time.*

*Proof*: Assume *A*_2_ is an Adversary who $\left(t^{\prime },q_{v},\varepsilon^{\prime }\right)$-compromises the EUF-CVA security of the e-voting scheme, and $A_{1}=A_{APKE}$ is the Adversary who $\left(t,q_{a},\varepsilon \right)$-compromises the unforgeability security of the APKE scheme. We aim to demonstrate that APKE scheme is not $\left(t,q_{a},\varepsilon \right)$-secure by showing how *A*_1_ can utilise *A*_2_ to $\left(t,q_{a},\varepsilon \right)$-compromise the unforgeability security of APKE scheme. In this scenario, *A*_1_ runs *A*_2_ as a subroutine while simulating its attack environment.

The APKE Challenger gives $\left(Params,pk_{e},sk_{e}\right)$ to *A*_1_. These keys $\left(pk_{e},sk_{e}\right)$ are generated from the PKE scheme. We allow *A*_1_ to possess the $\left(pk_{e},sk_{e}\right)$ of the PKE scheme, enabling it to simulate the Vote and Tally oracles for *A*_2_. Although *A*_1_ has access to $\left(pk_{e},sk_{e}\right)$ from the PKE scheme, this does not provide an advantage in breaking the unforgeability security. After this, *A*_1_ gives *Params* to *A*_2_ and completes the Registration phase.

During the Training phase, *A*_2_ sends a vote query {v,∅} to *A*_1_ which represents the Vote oracle from *A*_2_’s perspective. Here, *A*_1_ takes m=(v,∅), encrypts *m* to generate $\left(Trc,rand_{v}\right)$ to obtains *C*, and creates π for *C*. *A*_1_ then returns the result $\{Bal,Trc,rand_{v}\}=\alpha$ to *A*_2_. After that, *A*_2_ submits a tally query as *Bal* to *A*_1_. At this point, *A*_1_ assigns α=Bal and uses the Decrypt oracle to simulate the tallying process for *A*_2_. Specifically, *A*_1_ passes α to the Decrypt oracle to check its validity. The oracle provides the decryption result and *A*_1_ retrieves the validation result from the decryption result and returns the validation result whether it is invalid or valid to *A*_2_.

Eventually, *A*_2_ concludes the Training phase and initiates the Forging phase. During this phase, *A*_2_ forges Bal^*^ and issues Bal^*^ as its answer to *A*_1_, *A*_1_ assigns $\alpha^{*}=Bal^{*}$ and takes *A*_2_’s guess as its own guess. With a probability ε ′ ≤ n ( k ), *A*_2_ correctly forges Bal^*^. Since Bal^*^ is valid, then $\alpha^{*}$ is valid, *A*_1_ successfully compromises the unforgeability security.

Given that *A*_1_ accurately simulates the environment, we conclude that $\varepsilon =\varepsilon^{\prime }$ and $t=t^{\prime }$, as required. In this setup, *A*_1_ runs in time *t*, while *A*_2_ runs in time $t^{\prime }$. ◻

#### 6.2.4 Coercion-resistance.

**Theorem 5.** *Let PKE = {PKE.KeyGen, PKE.Encrypt, PKE.Decrypt}, Commitment = {Setup, Commit, Open} and* Σ* - protocol = {Commit, Challenge, Response} represent the secure public key encryption scheme, secure commitment scheme and secure* Σ*-protocol respectively. Let e-voting = {Register, Vote, Tally} represent the e-voting scheme. If the underlying PKE scheme is IND-CCA, Commitment satisfies binding property and* Σ*-protocol satisfies special honest-verifier zero-knowledge property, then the e-voting scheme satisfies coercion-resistance.*

*Proof*: Lemmas 1, 2 and 3 prove Theorem 5. ◻

**Lemma 1.** *Let PKE = {PKE.KeyGen, PKE.Encrypt, PKE.Decrypt} represent the secure public key encryption scheme. Let e-voting = {Register, Vote, Tally} represent the e-voting scheme. If the underlying PKE scheme is* $\left(t,q_{e},\varepsilon \right)$*-IND-CCA, then the e-voting scheme is* $\left(t^{\prime },q_{v},\varepsilon^{\prime }\right)$*-coercion-resistance, where*

t=t′,qe=qv,ε=ε′≤12+n(1λ) (9)

*Here,* $q_{v}$ *represents the number of vote queries, q*_*e*_ *represents the number of encrypt queries,* ε *represents the non-negligible advantage in breaking IND-CCA in PKE,* $\varepsilon^{\prime }$ *represents the non-negligible advantage in breaking coercion-resistance in e-voting, n represents a negligible function parameterised by* $1^{\lambda }$*, and t represents the attack completion time.*

*Proof*: Theorem 2 proved that if there exists a secure PKE scheme that is IND-CCA, then there exists a secure e-voting scheme that is IND-CBAA. From Theorem 5, if the underlying PKE scheme is IND-CCA, then the e-voting scheme satisfies coercion-resistance. This completes the proof. ◻

**Lemma 2.** *Let Commitment = {Setup, Commit, Open} represent the secure commitment scheme with binding property. If the underlying Commitment is* $\left(t,q_{com},\varepsilon \right)$*-binding, then the e-voting scheme is* $\left(t^{\prime },q_{v},\varepsilon^{\prime }\right)$*-coercion-resistance, where*

t=t′,qcom=qv,ε=ε′≤12+n(1λ) (10)

*Here,* $q_{v}$ *represents the number of vote queries, q*_*com*_ *represents the number of commitment queries,* ε *represents the non-negligible advantage in breaking binding property in Commitment,* $\varepsilon^{\prime }$ *represents the non-negligible advantage in breaking coercion-resistance in e-voting, n represents a negligible function parameterised by* $1^{\lambda }$, *and t represents the attack completion time.*

*Proof*: Assume *A*_2_ is an Adversary who $\left(t^{\prime },q_{v},\varepsilon^{\prime }\right)$-compromises the coercion-resistance security of the e-voting scheme, and $A_{1}=A_{Com}$ is the Adversary who $\left(t,q_{Com},\varepsilon \right)$-compromises the binding property of the Commitment scheme. We aim to demonstrate that Commitment scheme is not $\left(t,q_{Com},\varepsilon \right)$-secure by showing how *A*_1_ can utilise *A*_2_ to $\left(t,q_{Com},\varepsilon \right)$-compromise the binding property of Commitment scheme. In this scenario, *A*_1_ runs *A*_2_ as a subroutine while simulating its attack environment.

The Commitment Challenger gives $\left(Params,pk_{e},sk_{e}\right)$ to *A*_1_. These keys $\left(pk_{e},sk_{e}\right)$ are generated from the PKE scheme. We allow *A*_1_ to possess the $\left(pk_{e},sk_{e}\right)$ of the PKE scheme, enabling it to simulate the Vote and Tally oracles for *A*_2_. Although *A*_1_ has access to $\left(pk_{e},sk_{e}\right)$ from the PKE scheme, this does not provide an advantage in breaking the coercion-resistance security. After this, *A*_1_ gives *Params* to *A*_2_ and completes the Registration phase.

During the Training phase, *A*_2_ sends a vote query {v,coerc} to *A*_1_ which represents the Vote oracle from *A*_2_’s perspective. Here, *A*_1_ takes m=(v,coerc), computes a commitment *cmt* and corresponding opening value $rand_{v}$ on *m*, encrypts *m* to generate $\left(Trc,rand_{v}\right)$ to obtains *C*, and creates π for *C*. *A*_1_ then returns the result $\{Bal,Trc,rand_{v}\}=\alpha$ to *A*_2_. After that, *A*_2_ submits a tally query as *Bal* to *A*_1_. At this point, *A*_1_ assigns α=Bal and uses the Decrypt oracle to simulate the tallying process for *A*_2_. Specifically, *A*_1_ passes α to the Decrypt oracle to check its validity. The oracle provides the decryption result and *A*_1_ retrieves the validation result from the decryption result and returns the validation result whether it is invalid or valid to *A*_2_.

Eventually, *A*_2_ concludes the Training phase and initiates the Identification phase. During this phase, *A*_2_ issues $\left\{v^{*},coerc^{*}\right\}$ to *A*_1_. *A*_1_ assigns $m_{b}=v^{*}$, where b∈{0,1} and only one of them contained v^*^. *A*_1_ computes *cmt* and corresponding opening values *rand*_*b*_ for the same *cmt*. *A*_1_ obtains *C*_*b*_ by encrypting m^*^ and generates $\pi_{b}$ for *C*_*b*_. *A*_1_ returns $b=\{cmt,m_{0},rand_{0},C_{0},\pi_{0}\}$ and b′={cmt,m1,rand1,C1,π1} as the challenge to *A*_2_. With a probability ε′≤n(k), *A*_2_ can identify that $m_{0}\neq m_{1}$ and verification of b , b ′ return true. *A*_1_ takes *A*_2_’s guess as its own guess. Since $m_{0}\neq m_{1}$ and b , b ′return true, *A*_1_ successfully compromises the coercion-resistance security.

Given that *A*_1_ accurately simulates the environment, we conclude that $\varepsilon =\varepsilon^{\prime }$ and $t=t^{\prime }$, as required. In this setup, *A*_1_ runs in time *t*, while *A*_2_ runs in time $t^{\prime }$. ◻

**Lemma 3.** *Let* Σ-*protocol = {Commit, Challenge, Response} represent the secure* Σ-*protocol with special honest-verifier zero-knowledge property. If the underlying* Σ-*protocol is* $\left(t,q_{\Sigma },\varepsilon \right)$-*special honest-verifier zero-knowledge, then the e-voting scheme is* $\left(t^{\prime },q_{v},\varepsilon^{\prime }\right)$-*coercion-resistance, where*

t=t′,qΣ=qv,ε=ε′≤12+n(1λ) (11)

*Here,* $q_{v}$ *represents the number of vote queries,* $q_{\Sigma }$ *represents the number of challenge queries,* ε *represents the non-negligible advantage in breaking the special honest-verifier zero-knowledge in* Σ-*protocol,* $\varepsilon^{\prime }$ *represents the non-negligible advantage in breaking coercion-resistance in e-voting, n represents a negligible function parameterised by* $1^{\lambda }$, *and t represents the attack completion time.*

*Proof*: Assume *A*_2_ is an Adversary who $\left(t^{\prime },q_{v},\varepsilon^{\prime }\right)$-compromises the coercion-resistance security of the e-voting scheme, and $A_{1}=A_{\Sigma }$ is the Adversary who $\left(t,q_{\Sigma },\varepsilon \right)$-compromises the special honest-verifier zero-knowledge of the Σ-protocol. We aim to demonstrate that Σ-protocol is not $\left(t,q_{\Sigma },\varepsilon \right)$-secure by showing how *A*_1_ can utilise *A*_2_ to $\left(t,q_{\Sigma },\varepsilon \right)$-compromise the special honest-verifier zero-knowledge of Σ-protocol. In this scenario, *A*_1_ runs *A*_2_ as a subroutine while simulating its attack environment.

The Σ-protocol Challenger gives $\left(Params,pk_{e},sk_{e}\right)$ to *A*_1_. These keys $\left(pk_{e},sk_{e}\right)$ are generated from the PKE scheme. We allow *A*_1_ to possess the $\left(pk_{e},sk_{e}\right)$ of the PKE scheme, enabling it to simulate the Vote and Tally oracles for *A*_2_. Although *A*_1_ has access to $\left(pk_{e},sk_{e}\right)$ from the PKE scheme, this does not provide an advantage in breaking the coercion-resistance security. After this, *A*_1_ gives *Params* to *A*_2_ and completes the Registration phase.

During the Training phase, *A*_2_ sends a vote query {v,coerc} to *A*_1_ which represents the Vote oracle from *A*_2_’s perspective. Here, *A*_1_ takes m=(v,coerc), encrypts *m* to generate $\left(Trc,rand_{v}\right)$ to obtains *C*, and creates π for *C*, and runs Σ-protocol to obtain transcript (*a*,*e*,*z*) on public input x={v,C}. *A*_1_ then returns the result $\{Bal,Trc,rand_{v}\}=\alpha$ to *A*_2_. After that, *A*_2_ submits a tally query as *Bal* to *A*_1_. At this point, *A*_1_ assigns α=Bal and uses the Decrypt oracle to simulate the tallying process for *A*_2_. Specifically, *A*_1_ passes α to the Decrypt oracle to check its validity. The oracle provides the decryption result and *A*_1_ retrieves the validation result from the decryption result and returns the validation result whether it is invalid or valid to *A*_2_.

Eventually, *A*_2_ concludes the Training phase and initiates the Identification phase. During this phase, *A*_2_ issues $\left\{v^{*},coerc^{*}\right\}$ to *A*_1_. *A*_1_ computes C^*^, generates $\pi^{*}$ on C^*^, and runs Σ-protocol to receive transcript (a^*^,e^*^,z^*^) on $x=\{v^{*},C^{*}\}$. *A*_1_ returns $x=\{v^{*},C^{*}\}$ and (a^*^,e^*^,z^*^) as the real view to *A*_2_. Based on the special honest-verifier zero-knowledge of Σ-protocol, if a simulator exists that takes *x* and random challenge *e*, it can produce a simulation view (a valid transcript $\left(a^{\prime },e^{\prime },z^{\prime }\right)$) that is indistinguishable to (equal as probability distributions) the real view (a^*^,e^*^,z^*^) with a probability ε′≤n(k), *A*_2_ can simulate same view as *A*_1_’s view, *A*_1_ uses *A*_2_’s answer as its guess. Since the simulation view $\left(a^{\prime },e^{\prime },z^{\prime }\right)$ is indistinguishable from real view (a^*^,e^*^,z^*^), *A*_1_ successfully compromises the coercion-resistance security.

Given that *A*_1_ accurately simulates the environment, we conclude that $\varepsilon =\varepsilon^{\prime }$ and $t=t^{\prime }$, as required. In this setup, *A*_1_ runs in time *t*, while *A*_2_ runs in time $t^{\prime }$. ◻

#### 6.2.5 Cast-As-Intended (CAI) verifiability.

**Theorem 6.** *The coercion-resistant e-voting scheme proposed above has CAI verifiability.*

*Proof*: From Theorem 1, it is proven that IND-CVA implies IND-CAI, and the proposed scheme is secure against IND-CVA as proven in Theorem 3. So, the proposed scheme is IND-CAI secure. ◻

### 6.3 Correctness

The correctness of our e-voting scheme follows from the correctness of its cryptographic building blocks:

1. Public Key Encryption (PKE): Given a correctly generated key pair $\left(pk_{e},sk_{e}\right)$, decryption recovers the original vote.
2. Multi-Signature Scheme (MS): The signature σ on *C*_*i*_ is valid if and only if it is produced by all signers, ensuring that ballots are verifiably issued by the election committee.
3. Commitment Scheme: The binding and hiding properties ensure that the committed value cannot be altered or revealed prematurely.
4. Σ-Protocol: The transcript (*a*,*e*,*z*) guarantees that the voter correctly follows the proof of knowledge protocol without revealing *r*.
5. EOLTAA Scheme: The authentication proof $\pi_{i}$ ensures that only registered voters can submit ballots, and the linking mechanism prevents duplicate voting.
6. Final Tally: The correctness of decryption and verification ensures that all votes are accurately counted, and the zero-knowledge proof guarantees correctness without revealing private information. Specifically, the **Tally** phase ensures that all valid votes are included in the final election result:
  **Verification:** The election committee verifies each ballot using $\Phi_{\cdot }\text{Verify}$ to check its validity and $\Phi_{\cdot }\text{Link}$ to detect double votes. Any invalid or duplicate votes are discarded.
  **Decryption:** The election committee uses $PKE.\text{Decrypt}(sk_{e},C_{i})$ to decrypt each valid ciphertext *C*_*i*_, recovering the original vote *v* and randomness *r*. The correctness of *PKE* guarantees that decryption is consistent with encryption:
    $$PKE.\text{Decrypt}(sk_{e},PKE.\text{Encrypt}(pk_{e},(v,r)))=(v,r).$$ (12)
  **Result Computation:** The election result result is computed by aggregating the decrypted votes. The correctness of the multi-signature scheme ensures that the signature σ for each vote was valid, confirming the integrity of the decryption process.
  **Zero-Knowledge Proof:** The election committee generates a zero-knowledge proof $\pi_{\text{result}}$ to demonstrate the correctness of the tally process without revealing sensitive information.
    Thus, the final election result $\left(\text{result},\pi_{\text{result}}\right)$ satisfies public verifiability, ensuring that any third party can verify the accuracy of the tally without compromising voter privacy.

This correctness guarantees that the proposed e-voting scheme operates securely and reliably.

## 7 Result

Table 1 shows the comparison analysis of our scheme with the existing coercion-resistant e-voting schemes while Table 2 shows the comparison on the computation cost of our scheme compared to the existing coercion-resistant e-voting schemes.

**Table 1:** Comparison analysis of the existing coercion-resistance e-voting schemes.

Scheme	Security Property	Hardness	Model	Contribution and relationships between the Existing Schemes	Cryptographic Tools	Mechanism and Coercion Discovery
Smyth (2019) “Athena"	Individual verifiability, universal verifiability, privacy, unforgeability, coercion-resistance	DDH	Random oracle	Proposed a verifiable, coercion-resistant e-voting with linear complexity in JCJ setting	Homomorphic PKE, sigma protocol	Detection
Grontas *et al*. (2019)	Verifiability, eligibility, privacy, everlasting privacy, coercion-resistance	DDH	Random oracle	Combined everlasting privacy with JCJ coercion-resistance setting	Modified ElGamal cryptosystem, non-interactive zero-knowledge proofs of knowledge, verifiable Shuffles, blind signatures, plaintext equivalence test	Detection
Estaji *et al*. (2020)	Privacy, coercion-resistance, verifiability	DDH	Random oracle	Revisited the JCJ e-voting scheme [5] to enhance its usability and practicality	Paillier encryption, sigma protocol, zero-knowledge proofs	Detection
Lueks *et al*. (2020) “VOTEAGAIN”	Ballot privacy, coercion-resistance, verifiability	DDH	Random oracle	First revoting scheme capable of managing elections with millions of voters	ElGamal encryption, signature scheme, mixnet, zero-knowledge proofs	Detection
Cortier *et al*. (2022)	Coercion-resistance, vote privacy, verifiability	DDH	Random oracle	Highlight weakness of JCJ, fixed weakness in Juels *et al*.’s (2005) scheme	ElGamal encryption, zero-knowledge proofs, distributed key generation / threshold decryption, verifiable decryption mixnets, logical operations on encrypted bits, sorting encrypted data	Detection
Haines *et al*. (2023)	Ballot privacy, coercion-resistance, verifiability	DDH	Random oracle	Introduced a variant of VOTEAGAIN that reduces reliance on voting authorities	ElGamal encryption, signature scheme, mixnet, zero-knowledge proofs	Detection
Finogina and Herranz (2023)	CAI verifiability, coercion-resistance	DDH	Random oracle	Formalised security definitions that encompass both coercion-resistance and CAI verification, without relying on secure delivery channels setting between the election authority and voters	Sigma protocol, commitment scheme, homomorphic public key encryption	Prevention
Aranha *et al*. (2023)	Coercion-resistance	DDH	Random oracle	Improved Cortier *et al*.’s (2022) scheme, improved version of Chide, reduce complexity, faster tallying phase	ElGamal encryption (IND-CPA), designated-verifier zero-knowledge proof, circuits over encrypted bits, distributed random bit generation, verifiable mixnet	Detection
Chen *et al*. (2024)	Coercion-resistance, fairness, eligibility, uniqueness, anonymity, verifiability	DLOG	Random oracle	Proposed an e-voting scheme aimed to prevent bribery and coercion.	Mix-net, blind signature with sublimal channel, homomorphic encryption, smart cards	Detection
Our scheme	CAI verifiability, coercion-resistance, confidentiality, anonymity, unforgeability, double-voting prevention	DDH	Random oracle	Achieve CAI verifiability in coercion-resistance setting along with confidentiality, anonymity, unforgeability and double-voting prevention	Sigma protocol, commitment scheme, public key encryption, multi-signature, EOLTAA scheme	Prevention

**Table 2:** Comparison on the computation cost of existing coercion-resistant e-voting schemes.

Scheme	Computation Cost	
	Vote	Tally
Smyth (2019)	𝒪(log*n*)	𝒪(n)
Grontas *et al*. (2019)	𝒪(n)	𝒪(nlog*n*)
Estaji *et al*. (2020)	𝒪(n)	𝒪(nlog*n*)
Lueks *et al*. (2020)	𝒪(nlog*n*)	𝒪(nlog*n*)
Cortier *et al*. (2022)	𝒪(nlog*n*)	𝒪(nlog*n*)
Haines *et al*. (2023)	𝒪(n)	𝒪(nlog*n*)
Finogina and Herranz (2023)	𝒪(log*n*)	N/A
Aranha *et al*. (2023)	𝒪(nlog*n*)	𝒪(nlog*n*)
Chen *et al*. (2024)	𝒪(n)	𝒪(n)
Our Scheme	𝒪(n)	𝒪(n)

Note: N/A represents not provided

We observed that our scheme provided the most comprehensive security requirements, incorporating CAI verifiability within a coercion-resistance setting, as shown in Table 1. Most of the coercion-resistance schemes proposed in the literature conflict with CAI verifiability, as discussed in the Introduction. Another distinctive feature of our scheme is that, despite offering the richest security requirements, the computational cost for the Vote and Tally algorithms remains linear (𝒪(n)). Furthermore, our scheme ensured robust in-protocol coercion-resistance, which is achieved through prevention rather than detection. Prevention is more effective as it proactively addresses coercion, eliminating the problem before it occurs, and safeguarding voters’ freedom and autonomy from the start. In contrast, detection only addresses coercion after it has already taken place. Prevention mechanisms can be integrated directly into the system design, ensuring that the voting process is secure from the outset. Detection mechanisms, however, typically rely on monitoring or auditing, which may not always be effective and are prone to loopholes or false negatives. Additionally, prevention is often more efficient in the long term, as it avoids the need for complex detection and auditing processes. Detection systems may require extensive surveillance, legal processes, and additional resources to identify and act on instances of coercion.

In addition, we observed that the proposed scheme by Chen *et al*. [17] has the same computational cost for the Vote and Tally algorithms as our proposed scheme from Table 2. The main difference between our scheme and Chen *et al*.’s [17] is that we incorporate CAI verifiability in our Vote algorithm, whereas their scheme does not satisfy CAI verifiability in the coercion-resistant setting. Additionally, our scheme offers a more robust in-protocol coercion-resistance. We primarily highlight the significant differences between our scheme and Chen *et al*.’s [17] in terms of the voter’s role, the approaches to achieving coercion-resistance and technical mechanisms employed, and the overall security models and assumptions as follows:

The Voter’s Role in Coercion-Resistance:

- In Chen *et al*.’s [17] coercion-resistant protocol, the voter actively participates by embedding subliminal information (PIN and subliminal messages) to resist coercion. However, this approach requires the voter to take action after being coerced, and the coercion is detected after the voting phase.
- In our proposed scheme, the voter’s interaction is fully secure during the voting process itself. The cryptographic protocol ensures that even if the voter is coerced, they cannot reveal their vote to the coercer, thus preventing coercion from occurring in the first place.

Mechanism and Coercion Discovery (Detection vs. Prevention):

- Chen *et al*. [17] focuses on detecting coercion after the voting process. The AAC analyses the subliminal message during the dispute phase to uncover the true intent of the voter. Coercion is not directly prevented during the voting phase, but if coercion is suspected, it can be revealed after the ACC analyses the subliminal messages.
- Our scheme focuses on preventing coercion during the voting process. We build coercion-resistant directly into the voting protocol through cryptographic commitments, multi-signature verification, and zero-knowledge proofs, the voter’s interaction with the election committee is secure, and the voter cannot be forced to reveal a verifiable vote, making coercion much harder to execute.

Security Models and Assumptions:

- Chen *et al*. [17] assumes the presence of a trusted AAC that will act during a post-election phase to resolve disputes using subliminal messages. It also relies on subliminal communication channels for the voter to transmit additional covert information about their true vote.
- Our proposed scheme is based on the hardness of the cryptographic assumptions. The voter’s choices are hidden through commitments and zero-knowledge proofs, making it impossible for an adversary (coercer) to extract or force the voter’s true choice during or after the protocol.

## 8 Conclusion

We presented a provably secure coercion-resistant e-voting scheme and proved that our proposed e-voting scheme possesses the security properties of confidentiality, anonymity, unforgeability, and CAI verifiability with asymptotic complexity of *O*(*n*). We also proved that anonymity implies CAI verifiability for e-voting schemes. By enhancing the security and privacy guarantees of the existing e-voting scheme, we believe that we address the shortcomings of the current system and our comprehensive security models can be a useful reference for the design of future e-voting schemes.

The limitation of this work is that it focuses solely on the interactive communication between the election committee and the voter during the execution of the voting protocol, ensuring that the encryption of the voter’s choice accurately reflects their intent. The development of a non-interactive voting protocol that also achieves coercion-resistance remains an open challenge and is left as a direction for future research. Besides that, our proposed coercion-resistant e-voting scheme could be extended to satisfy post-quantum security requirements. Current public key encryption schemes are vulnerable to adversaries equipped with quantum computing capabilities. Lattice-based cryptography appears to be the most promising approach to address this challenge. For instance, a suitable public key encryption scheme could be constructed using the Ring Learning With Errors (RLWE) problem. The sigma protocol could be replaced with a lattice-based zero-knowledge interactive system, while a lattice-based commitment scheme offers a strong candidate for a quantum-safe commitment mechanism. However, these suggestions serve only as a foundational concept for a post-quantum coercion-resistant e-voting scheme. A more in-depth investigation would be required to fully explore and realise this idea. Additionally, we plan to implement the proposed scheme to assess its practical performance and computational efficiency in real-world scenarios.
